# Biobank for craniosynostosis and faciocraniosynostosis, rare pediatric congenital craniofacial disorders: a study protocol

**DOI:** 10.1007/s00381-024-06555-w

**Published:** 2024-08-12

**Authors:** Lucia De Martino, Peppino Mirabelli, Lucia Quaglietta, Ursula Pia Ferrara, Stefania Picariello, Domenico Vincenzo De Gennaro, Marco Aiello, Giovanni Smaldone, Ferdinando Aliberti, Pietro Spennato, Daniele De Brasi, Eugenio Covelli, Giuseppe Cinalli

**Affiliations:** 1 Neurooncology Unit, Santobono-Pausilipon Children’s Hospital, AORN, Naples, Italy; 2 Clinical and Translational Research Unit, Santobono-Pausilipon Children’s Hospital, AORN, Naples, Italy; 3 IRCCS SYNLAB SDN, Naples, Italy; 4 Pediatric Neurosurgery Unit, Santobono-Pausilipon Children’s Hospital, AORN, Naples, Italy; 5 Clinical Genetic Unit, Santobono-Pausilipon Children’s Hospital, AORN, Naples, Italy; 6 Pediatric Neuroradiology Unit, Santobono-Pausilipon Children’s Hospital, AORN, Naples, Italy

**Keywords:** Craniosynostosis, Biobank, Rare pediatric disease, Congenital cranial malformation

## Abstract

**Purpose:**

Craniosynostosis (CRS) is a rare congenital cranial malformation in which 1 or more cranial or facial sutures are fused in utero or rapidly fused in early infancy. The cranial sutures separate the skull bone plates and enable rapid growth of the skull in the first 2 years of life, in which growth is largely dictated by growth of the brain. CRS is a rare disease that occurs in 1 in 2100 to 1 in 2500 births and may be either nonsyndromic (also referred to as isolated) or syndromic. In syndromic CRS, other birth defects are present next to the CRS. The distinction between nonsyndromic and syndromic manifestations is made on the basis of dysmorphologic evaluation and genetic evaluation. Owing to advances in genetic diagnostics, nonsyndromic patients are increasingly recognized as syndromic patients. CRS treatment is almost entirely surgical and is sometimes paired with postoperative helmet therapy for maintenance. Corrective procedures are complex, long, and associated with the risk of numerous complications, including heavy blood loss and its sequelae. Although surgery may restore a normal appearance, even in nonsyndromic patients, patients may experience persistent deficits in intellectual ability and cognitive function. The European Commission (EC) has prioritized rare diseases in recent horizon European research programs; indeed, collections or even individual samples may be extremely valuable for research.

**Methods and results:**

Here, we present a study protocol in which the combined expertise of clinicians and researchers will be exploited to generate a biobank dedicated to CRS. The generation of the CRS biobank presented in this study will include the collection of different types of biological materials as well as advanced radiological images available to the scientific community.

**Conclusion:**

The activation of a CRS biobank will provide an opportunity to improve translational research on CRS and to share its benefits with the scientific community and patients and their families.

## Introduction

Craniosynostosis (CRS) is a rare congenital cranial malformation that may affect one or more sutures of the cranial vault, of the maxillofacial skeleton, or of the synchondrosis of the cranial base. More frequently, one or more cranial sutures can already be absent at birth, but in less frequent syndromes, they may fuse precociously in the earliest months of life together with sutures and synchondrosis of the maxillofacial skeleton and cranial base. The cranial vault sutures separate the skull bone plates and enable rapid growth of the skull in the first 2 years of life, in which growth is largely dictated by growth of the brain. Cranial sutures are essential for skull growth in the first 2 years (the period of rapid brain growth). Thereafter, the process of appositional growth and internal resorption of the skull is the major process by which the skull increases in size. Premature fusion of cranial sutures impedes normal growth of the skull, resulting in characteristic anatomic malformations of the skull. Craniosynostosis occurs in 1 in 2100 to 1 in 2500 births and may be either nonsyndromic (also referred to as isolated) or syndromic. In syndromic CRS, other birth defects are present next to the CRS. In syndromic CRS, more than 1 cranial suture is usually prematurely fused. The distinction between nonsyndromic and syndromic manifestations is made on the basis of dysmorphologic evaluation and genetic evaluation. Owing to advances in genetic diagnostics, nonsyndromic patients are increasingly recognized as syndromic patients. The discovery of the P250R mutation in the FGFR3 gene in patients with unilateral bilateral coronal suture synostosis clearly illustrates this phenomenon. Approximately 60% of all craniosynostoses are of the nonsyndromic type, and approximately 40% are of the syndromic type. Among nonsyndromic craniosynostoses, sagittal suture synostosis is the most common, followed by metopic suture synostosis. Recently, the prevalence of metopic suture synostosis has clearly risen both in Europe and the United States, but this has not been explained. Synostosis of 1 or both lambdoidal sutures is very rare. Among the syndrome types, Muenke syndrome is the most common, followed by Crouzon syndrome and Pfeiffer syndrome (1).

Human biobanks collect all types of biological samples, such as blood, tissue, cell or DNA, and/or related data, such as associated clinical and research data, as well as biomolecular resources, including model and microorganisms, which might contribute to the understanding of the physiology and disease status of humans. The European Commission (EC) has prioritized rare diseases in recent horizon European research programs; indeed, collections or even individual samples may be extremely valuable for research (2). Here, we present a study protocol in which the combined expertise of clinicians and researchers will be exploited to generate a biobank dedicated to CRS. The generation of the CRS biobank presented in this study will include the collection of different types of biological materials as well as advanced radiological images available to the scientific community.

## Methods

### General aim

Based on a 2-year follow-up prospective cohort of patients with CRS, our study aimed to generate a CRS biobank to collect biological samples, imaging data, and patient data based on an innovative and easily accessible bioresource for a comprehensive study of this rare disease.

The objectives are as follows:

I. To implement the diagnosis and follow-up of children affected with craniosynostosis. A trained multidisciplinary team (physicians, nurses, pharmacists, technicians, and other health professionals) will be dedicated to the management of these children. All patient data will be associated with biological samples and images to generate a comprehensive biobank.
II. To develop a comprehensive biobanking service (clinical data, imaging, and biosamples) for CRS patients to collect biological material and advanced medical imaging data. The biobank will comply with the ISO 20387 biobanking international standards and will be included in the most important national and international biobanking infrastructure, such as BBMRI.it, BBMRI-ERIC, and UNIAMO (the Italian Federation of Rare Diseases). The aim of this study is to identify a state-of-the-art and easily findable bioresource dedicated to the scientific community for studying CRS.
III. To develop data curation and analytics tools for the integration of imaging and genomic data and the development of innovative systems based on artificial intelligence and machine learning approaches to support clinical decisions. The genomic analysis will include a comprehensive profile for most of the known genes mutated in the case of CRS (i.e., FGFR-1, -2, RUNX1, and TWIST-1). In parallel, imaging analysis will be performed by skilled researchers working in units 1 and 2 to define radiomic features to be evaluated as noninvasive biomarkers.

### Study design and population

Our study is a monocentric retrospective/prospective cohort study that included approximately 360 patients affected by CRS treated at Santobono-Pausilipon Hospital in Naples, Italy. The Santobono-Pausilipon Hospital is a reference center for the treatment of pediatric diseases in southern Italy with high expertise in the management of CRS (3–13) and has a scientific agreement with the IRCCS Synlab SDN SpA.

Inclusion criteria:

- Patients aged less than 14 years at diagnosis
- Diagnosis of CRS
- Parents agree to participate in the study by signing informed consent

Exclusion criteria:

- Age more than 14 years at diagnosis
- Diagnosis of positional cranial deformity
- Parents disagree to participate

### Study procedures

**Fig. 1:**
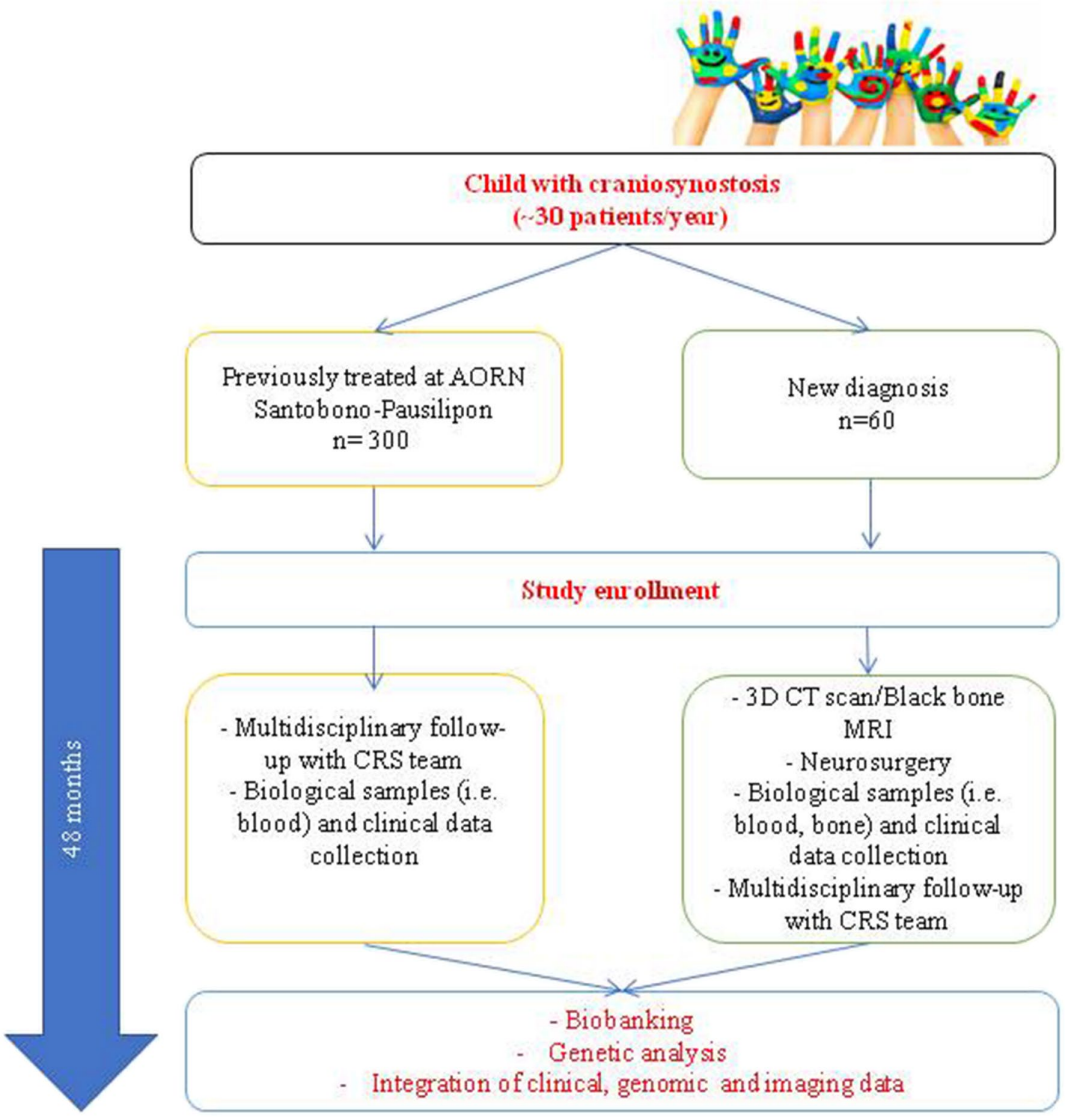
Flow chart of the “Biobank for Craniosynostosis and Faciocraniosynostosis: a Rare Pediatric Congenital Craniofacial Disorders” research study funded by the National Recovery and Resilience Plan (NRRP)

The study flow chart is presented in Fig. 1. The plan is to include 360 patients treated for 2 years (concordant with the number of patients treated yearly at Santobono-Pausilipon Hospital) divided into two subgroups:

- Retrospective cohort including approximately 300 (30 pts/year) children and adolescents with CRS (diagnosis 2010–2022)
- Prospective cohort including approximately 60 new CRS-diagnosed infants

Our team, consisting of multiple different specialists, will perform multidisciplinary evaluations of all children and adolescents (Table 1). Biological samples will encompass bone fragments (when feasible, in accordance with surgical protocols), plasma, mononuclear cells, and whole blood. All samples will undergo cryopreservation either in the vapor phase of liquid nitrogen or in mechanical freezers set at − 80 °C. Informed consent from parents was obtained from the local ethical committee. To ensure the integrity of the samples, the storage period was set to a maximum of 10 years.

**Table 1:** List of craniofacial team members at Santobono-Children’s Hospital

Professional team members	Role in the project
Neurosurgeons	-Operative correction of cranial/facial deformities -Trepanning with/without reconstruction; analysis and therapy of brain malformations
Oral and maxillofacial surgeons	Dentofacial deformities assessment
Neuroradiologists	-Assessment of the imaging tests -Helping in the select the optimal treatment
Ophthalmologists	Evaluation of vision, refraction, and motility abnormalities
Geneticists	Analysis of congenital defects and heredity: gene/chromosome analysis
Otolaryngologists	Evaluation of obstructive sleep apnea syndrome, hearing impairments
Nurses	Care of patients and sample collection
Dentist/orthodontists	Dental deformities assessment
Occupational/physical therapists	Neuro-rehabilitation
Pediatricians	Analysis of general health, growth, and development in children
Psychologists	-Psychometric/psychologic screening -Treatment/guidance and social family counseling
Speech and language pathologists	Speech and breathing analysis and treatment

Genetic analysis will be performed according to practice guidelines (1):

- In children with proven CRS and an evident phenotype, we will offer targeted clinical genetic diagnostics.
- In children with proven CRS and birth defects and/or developmental disorders, we will perform CGH array analysis, targeted DNA diagnostics or NGS trio analysis, possibly followed by exome opening.
- In children with proven CRS without an evident phenotype, we will perform an NGS CRS panel and trio analysis if indicated.

Radiological diagnosis and follow-up of CRS will be performed according to current clinical practice guidelines (1) as follows:

- Clinical assessment: CRS should be recognized in time for optimal treatment, excluding positional cranial deformity.
- Standard skull and face X-rays, which are traditionally considered pillars of diagnostic tools for CRS, have been progressively abandoned due to improvements in more recent complementary techniques.
- Ultrasound (diagnosis): ultrasound is a primary imaging modality for the detection or exclusion of craniosynostosis in children up to the age of 8 to 9 months
- 3D low-dose computed tomography (CT) scan (preop, immediate postop, follow-up): using these low-dose techniques, the effective radiation dose is reduced to 0.02–0.05 mSv (comparable to the effective radiation dose of plain skull radiography ranging from 0.01 to 0.04 mSv), while adequate diagnostic image quality is maintained
- Magnetic resonance imaging (MRI) (preop, immediate postop, follow-up varying according to different types of CRS)
- Standard anatomical sequences
- Diffusion-weighted imaging for tractography and connectomics
- High-definition sequences for the CSF/brain interface
- Angio-MRI with venous sequences to ascertain the existence and degree of venous outflow resistance
- Cerebral perfusion MRI to ascertain the effects of craniofacial remodeling on brain perfusion
- Black bone MRI is a promising alternative to 3D CT scan of the skull in syndromic CRS for which an MRI examination to detect associated intracranial abnormalities is indicated.
- 3D optical scan (pre- and postoperative and follow-up): This technique enables the electronic capture of a high-resolution 3D model of the patient’s head shape in a few seconds, allowing us to follow the variation in shape for each patient over time.

In addition, we planned to include innovative in vitro biomarkers to evaluate neurological damage in CRS patients. Specifically, neurofilament light chain (NfL) and glial fibrillary acidic protein (GFAP) levels will be determined before and after surgery. Moreover, in the retrospective cohort, NfL and GFAP levels will be tested as support for monitoring neurological functions.

### Sample size calculation

During the last 3 years, the advanced neurosurgery unit of unit 1 included the following patients: 2019, with 36 patients (27 males, 9 females); 2020, with 39 patients (27 males and 12 females); and 2021, with 37 patients (28 males and 9 females). We expect to include in our biobank prospective collection of approximately 60 cases in 2 years and for each patient to dispose of sampling pre- and postsurgery. We considered a priori power analysis to calculate the sample size of the study. Then, we performed a test of a single proportion with two tails, considering an effect size of 0.3, an alpha error of 0.05, and a constant proportion of 0.5 having syndromic cases. Based on these assumptions, we estimated a minimum of 28 samples/year to achieve a power of 0.9. Moreover, for retrospective collection, we planned to examine approximately 300 clinical records and to call the CRS patients for long-term follow-up. Based on these assumptions, we performed a post hoc power analysis using two-tailed Fisher’s exact test with an alpha level of 0.05, and we determined that the total sample size of 300 patients was adequate to achieve a power above 0.9.

### Data collection

The following data will be collected from a dedicated electronic database: (i) patient identification number and demographics (year of birth, sex, ethnicity); (ii) disease characteristics, including subclass and subphenotype; (iii) patient medical history, comorbidity and diagnostic and radiological measurements (imaging, physical examination, laboratory data, etc.); (iv) risk and prognostic factors that are known to affect the onset or course of the disease; (v) treatment (therapy); (vi) details on family history (standardized family tree investigations or questions about disease occurrence in family members); (vii) prognosis and long-term outcomes; and (viii) genetic data, if available. Personal data will be collected only with explicit consent from individuals, clearly specifying the purposes of data collection and processing. Consent forms are designed to be comprehensive and transparent. The collected data will undergo pseudonymization processes by encryption to transform personal data into ciphertext that can only be decrypted with a specific key to prevent the identification of individuals unless strictly necessary for research purposes; they will be stored securely using encrypted databases and storage systems. Access to the data is restricted to the authorized personnel involved in the research project. The data will be retained for a maximum of 10 years (the same time for each biosample); after this period, the data will be securely deleted or anonymized. To protect personal data against unauthorized access, alteration, disclosure, or destruction, an implementation of security has been performed, including regular security audits and staff training on data protection practices.

### Planned analysis

Data processing activities will be conducted in accordance with defined research objectives and are limited to what is necessary for achieving those objectives. Processing activities are regularly reviewed and monitored. Categorical variables will be reported as numbers (percentages). Continuous nonparametric variables are presented as medians (interquartile ranges), and continuous parametric variables are presented as the means and standard deviations. Differences between groups will be compared by chi-square or Fisher’s exact tests for categorical variables and the Mann‒Whitney and Wilcoxon rank-sum tests for continuous nonparametric variables. Paired and unpaired *t* tests were used for continuous parametric variables. Statistical significance will be set at a *p* value <0.05.

### Timing for data analysis

The lifespan of the current project is 24 months, according to the National Recovery and Resilience Plan (NRRP) regulation. Since we plan to establish a biobank, the duration of its activities will not be limited to this period but will extend beyond the project’s lifespan. Clinical data, therapy, and follow-up data will be recorded and associated with each biosample.

## Results

The study started to recruit patients from May 2023. In our hospital, a multidisciplinary team takes care of patients with nonsyndromic or syndromic CRS, as the management of CRS is complex. Because CRS is rare, centralization of care is desirable, resulting in a maximum of expertise, ensuring a high quality of care, and permitting scientific research aimed at improving care. The fragility of children is a concern throughout the entire research process, making it ethically challenging for young people to participate in biobanking activities. Pediatric research activities raise more important ethical questions than those that apply to adults. Since our biobank involves children, we considered various ethical issues, such as (i) parents’ informed consent and children’s assent, (ii) data protection for minors, (iii) biological sample retention, (iv) greater discomfort and distress, and (v) the need to reconfirm consent when children become adults.

The pediatric biobank dedicated to the study of CRS planned in the present study provides a standardized collection of biological samples for sharing at the national and international levels. To this aim, the biobank has dedicated spaces (a cryogenic room with remote monitoring and a processing laboratory) and personnel (biobank manager, biologist, and technician) involved in sample processing and storage, applying standard operating procedures. To make the bioresource visible to the external user, we foresee including the CRS Biobank in the national node of BBMRI.it (https://www.bbmir.it) as well as in UNIAMO (the Italian Federation for Rare Diseases) (https://www.uniamo.org) and the European Joint Programme for Rare Diseases (EJP RD). Specifically, following the EJP RD recommendations, the most direct pathway to inclusion in the EJP-RD catalog is through the Italian national node of the BBMRI-ERIC (www.bbmr.it). In this context, we are actively working to finalize the establishment of our biobank in compliance with BBMRI.it regulations. This involves securing dedicated space, acquiring necessary instruments, and hiring personnel. Our institution, AORN Santobono-Pausilipon, has officially recognized the biobank, which will be overseen by a supervisor supported by quality assurance and an IT manager. This aligns well with our previously outlined plan to join the BBMRI network, further expanding our potential biobank access and research partnerships. A dedicated catalog will be created to present the biobank to the scientific community (obtaining a digital object identifier to cite and locate the bioresource) and external users. Dedicated software will be used for storing and tracking all biological material, including detailed aliquots/derivatives location and freezer management, sample request, shipment, and participant information. Then, the biobank will be subjected to an audit for ISO 9001:2015 accreditation to ensure consistency and standardization of practice.

Moreover, according to FAIR principles (https://www.ejprarediseases.org/fairification/), we have taken proactive steps to enhance our biobank’s compliance. To enhance this section, we will incorporate the guidelines provided by the European Joint Programme on Rare Diseases (EJP RD) for services, guidelines, tools, and training in FAIRification. These resources will help us to better harmonize and link up data from various sources, such as genomics archives, health records, patient registries, and biobanks.

To ensure that our biobank is “Findable”, we have authored this paper and have actively engaged in scientific meetings to showcase our efforts. In pursuit of making the biological samples “Accessible”, we are developing a dedicated website for the CRS biobank, catering to both professionals and the general public, providing comprehensive information and facilitating communication. To promote “interoperability”, our biobank team adhered to standardized operating procedures for sample processing. Additionally, we are working toward ISO 9001 certification and aligning with the ISO:20387 biobank standardization to further enhance our practices. Finally, to facilitate data reuse, we are evaluating specialized IT solutions, including a cloud-based platform such as the European Open Science Cloud (EOSC) (https://www.eosc-life.eu/) enabling the management, storage, and reuse of clinical data and digital MR images. Furthermore, to ensure compliance with the GDPR Regulation, we implemented a specific informed consent process tailored to the treatment of personal data, which was provided to parents. Our Data Protection Officer oversees GDPR compliance and serves as a point of contact for data subjects and authorities and policies. Additionally, all samples stored in our biobanks are pseudonymized, and the collected data are securely stored on IT servers to mitigate the risk of data breaches.

## Discussion

Rare diseases, also known as “orphan” or “neglected” diseases, are still underresearched worldwide. New diagnostic and therapeutic strategies are urgently needed to manage rare diseases, and biobanks are recognized as effective tools for advancing rare disease research. National and international biobanks for rare diseases are needed to bring patients together to facilitate research, as the number of patients in local jurisdictions for each rare disease is too low. Orphanet, an online catalog of over 6000 rare diseases and a directory of expert resources for participating countries, recently stated that rare disease biobanks are “the only way to pool data in order to achieve a sufficient sample size for epidemiological and/or clinical research”.

(https://www.orpha.net/orphacom/cahiers/docs/GB/Registries.pdf). The involvement of CRS in biobanking in children is particularly highlighted (2, 14). CRS biobanks can guarantee the long-term storage of samples from children affected with CRS for future research purposes. However, it is vital to encourage and maintain children’s engagement in medical research programs and biobanking projects, especially as children become adults, and reconsent procedures must be applied (15).

A recent evolution in biobanking is the development of imaging biobanks (16). Our group provided the first radiological evidence of the impact of craniofacial surgery on dural sinus anatomy and venous drainage (17). The venous anomalies described in patients with syndromic CRS are not static, and posterior cranial vault distraction plus foramen magnum decompression triggers a dynamic process that can lead to modifications of intracranial venous drainage (17). The traction exerted by the distracted bone flap onto the occipitoparietal dura mater adherent to the inner calvaria may account for the enlargement of the dural sinus throughout the distraction period. The impact of these modifications on venous pressure, intracranial pressure, hydrocephalus and long-term neurocognitive functioning remains to be determined. Similar changes are expected in other functional and anatomical parameters (arterial blood flow, tractography, and connectomics) that require advanced functional MRI technology, sequences, and postprocessing techniques.

The generation of a multidisciplinary CRS biobank represents an opportunity to improve translational research on CRS and to share the benefits with the scientific community and patients and their families.

## Data Availability

The data that support the findings of this study will be available on request from the corresponding author (LDM).

## Abbreviations

IRCCS: Istituto di Ricovero e Cura a Carattere Scientifico (Scientific Institute for Hospitalization and Care)
CRS: Craniosynostosis
EC: European Commission
CT: Computed tomography
MRI: Magnetic resonance imaging
NfL: Neurofilament light chain
GFAP: Glial fibrillary acidic protein
NRRP: National Recovery and Resilience Plan
