# Correction: Biobank for craniosynostosis and faciocraniosynostosis, rare pediatric congenital craniofacial disorders: a study protocol

**DOI:** 10.1007/s00381-024-06625-z

**Published:** 2024-09-23

**Authors:** Lucia De Martino, Peppino Mirabelli, Lucia Quaglietta, Ursula Pia Ferrara, Stefania Picariello, Domenico Vincenzo De Gennaro, Marco Aiello, Giovanni Smaldone, Ferdinando Aliberti, Pietro Spennato, Daniele De Brasi, Eugenio Covelli, Giuseppe Cinalli

**Affiliations:** 1 Neurooncology Unit, Santobono-Pausilipon Children’s Hospital, AORN, Naples, Italy; 2 Clinical and Translational Research Unit, Santobono-Pausilipon Children’s Hospital, AORN, Naples, Italy; 3 IRCCS SYNLAB SDN, Naples, Italy; 4 Pediatric Neurosurgery Unit, Santobono-Pausilipon Children’s Hospital, AORN, Naples, Italy; 5 Clinical Genetic Unit, Santobono-Pausilipon Children’s Hospital, AORN, Naples, Italy; 6 Pediatric Neuroradiology Unit, Santobono-Pausilipon Children’s Hospital, AORN, Naples, Italy

**Correction: Child's Nervous System**

10.1007/s00381-024-06555-w

The Funding information section was missing from this article and should have read 'This research (project code PNRR-MR1-2022–12376512) is funded by the National Recovery and Resilience Plan (NRRP) and by the Italian Ministry of health according to the Missione 6/componente 2/Investimento: 2.1 “Rafforzamento e potenziamento della ricerca biomedica del SSN”, finanziato dall’Unione europea – Next Generation EU, CUP H63C22000470006'.

The original article has been corrected.

